# Prognostic Significance and Treatment Response Associations of Genetic Mutations in Chronic Myelomonocytic Leukemia: A Retrospective Cohort Study

**DOI:** 10.3390/biomedicines12112476

**Published:** 2024-10-28

**Authors:** Jing Di, Leonard N. Yenwongfai, Talal Arshad, Bin Huang, Jaclyn K. McDowell, Eric B. Durbin, Reinhold Munker, Sainan Wei

**Affiliations:** 1 Department of Pathology & Laboratory Medicine, University of Kentucky, Lexington, KY 40536, USA; 2 Department of Pathology, The Johns Hopkins University School of Medicine, Baltimore, MD 21205, USA; 3 Division of Cancer Biostatistics, University of Kentucky, Lexington, KY 40536, USA; 4 Markey Cancer Center, Cancer Research Informatics Shared Resource Facility, University of Kentucky, Lexington, KY 40536, USA; 5 Kentucky Cancer Registry, Lexington, KY 40504, USA; 6 Division of Hematology, Blood and Marrow Transplantation, University of Kentucky, Lexington, KY 40536, USA; rmunker@uky.edu

**Keywords:** chronic myelomonocytic leukemia, genetic profiling, prognostic biomarkers, survival analysis

## Abstract

**Background:** This retrospective cohort study investigates the prognostic significance of genetic mutations in Chronic Myelomonocytic Leukemia (CMML) and their association with treatment responses among patients treated at a single institution, juxtaposed with a statewide dataset from Kentucky. **Methods:** The study includes 51 patients diagnosed with CMML under the World Health Organization criteria from January 2005 to December 2023. It examines their genomic profiles and subsequent survival outcomes. The analysis also categorizes patients into CMML-1 and CMML-2 subtypes and assesses survival differences between transformed and non-transformed cases. **Results:** Mutations in TET2, ASXL1, and SRSF2 were found to significantly influence survival, establishing their roles as critical prognostic markers. Additionally, the cohort from the University of Kentucky exhibited distinct survival patterns compared to the broader Kentucky state population, suggesting that demographic and treatment-related factors could underlie these variances. **Conclusions:** This research underscores the pivotal role of targeted genetic profiling in deciphering the progression of CMML and refining therapeutic strategies. The findings emphasize the necessity for advanced genetic screening in managing CMML to better understand individual prognoses and optimize treatment efficacy, thereby offering insights that could lead to personalized treatment approaches.

## 1. Introduction

Chronic Myelomonocytic Leukemia (CMML) is an enigmatic clonal hematopoietic disorder that bridges myelodysplastic syndromes (MDS) and myeloproliferative neoplasms (MPN), manifesting a considerable risk of progression to acute myeloid leukemia (AML) within one and three years of diagnosis. Recent advancements in the diagnostic criteria of CMML have increasingly recognized the importance of molecular features in determining the prognosis of affected patients [1]. CMML is notably challenging due to its varied clinical presentations and outcomes, which are profoundly influenced by genetic abnormalities and the demographic characteristics of the patient population [1,2].

CMML is typically categorized into two subtypes based on the percentage of bone marrow blasts: CMML-1, which has less than 10% blasts, and CMML-2, which has 10–19% blasts or evidence of transformation to AML. Further stratification considers the disease’s proliferative and dysplastic features [1]. The “proliferative” variant is characterized by higher white blood cell counts and an elevated risk of leukemic transformation, while the “dysplastic” variant exhibits more features of bone marrow failure. At the molecular level, mutations in genes such as TET2, ASXL1, and SRSF2 are frequently observed, and their presence is often linked to prognosis [3]. For instance, the ASXL1 mutation is associated with poorer outcomes and a higher risk of transformation to AML [1]. Understanding these variants is critical for personalized treatment strategies and improving patient outcomes.

Globally, research into CMML has revealed a spectrum of survival outcomes and responses to treatment, highlighting the role of geographic and ethnic factors in disease progression and management efficacy. Studies conducted across different regions—ranging from the United States to Europe and Asia—have documented significant disparities in median survival rates, which can be as varied as the populations themselves [3,4]. Such variations underscore the necessity for a deeper understanding of the biological and social determinants influencing CMML.

Kentucky, particularly the Appalachian region, is identified as a high-risk area for various cancers [2], suggesting a potential unique environmental or genetic interplay affecting disease characteristics and patient outcomes. Despite the progress in understanding the molecular underpinnings of CMML, patient outcomes remain dismal, with a median overall survival (OS) of only 17 months, as reported in a comprehensive population-based study from 2007–2011 [3].

Our study posits that Kentucky’s distinct socio-environmental and genetic backdrop might influence both the mutation spectrum and survival outcomes in CMML. Focusing on a cohort from this high-risk region, our single-center study aims to delineate the correlation between specific gene mutations, overall survival, and the risk of AML transformation. Through this focused exploration, we seek to uncover potentially divergent disease phenotypes and inform targeted therapeutic strategies that could enhance patient outcomes in specific subpopulations.

## 2. Materials and Methods

### 2.1. Patient Population

This retrospective cohort study included 51 patients diagnosed with CMML at a single institution between January 2005 and December 2023. Patients were eligible if they met the World Health Organization’s (WHO’s) fifth edition of diagnostic criteria for CMML, had comprehensive testing, and had follow-up data available. The study was approved by the Institutional Review Board of the University of Kentucky.

### 2.2. Data Collection

Clinical data, including patient demographics, disease characteristics, treatment regimens, and clinical outcomes, were extracted from electronic medical records. The key variables collected were age at diagnosis, sex, white blood cell count, percentage of peripheral blood monocytes, hemoglobin levels, platelet count, bone marrow blast percentage, and cytogenetic abnormalities. Treatment regimens were classified into broad categories, including azacitidine-based, decitabine-based, hydroxyurea-based, combination therapy, and supportive care.

### 2.3. Genomic Profiling

Genomic DNA was extracted from bone marrow aspirates or peripheral blood samples at diagnosis. Next-generation sequencing (NGS) was performed using a panel of 97 genes, including 50 common myeloid focus genes such as TET2, ASXL1, SRSF2, EZH2, and TP53, known to be mutated in CMML. Variants were identified and annotated using standard bioinformatics pipelines, and only variants with a variant allele frequency (VAF) of ≥5% were considered for analysis.

### 2.4. Survival Analysis

Survival data, including overall survival (OS) and progression-free survival (PFS), were collected. OS was defined as the time from diagnosis to death from any cause, and PFS was defined as the time from diagnosis to disease progression. Patients were censored at the last follow-up if neither event had occurred. Kaplan–Meier survival curves were generated to estimate OS and PFS, and differences between groups were assessed using the log-rank test.

The survival analysis also includes Kaplan–Meier survival estimates from a broader statewide cohort of 254 CMML cases from the Kentucky Cancer Registry from 2012 to 2021. This enhancement allows for a direct comparison of localized hospital-based outcomes with broader statewide trends, offering insights into the generalizability and applicability of our findings.

### 2.5. Statistical Analysis

The impact of specific gene mutations on survival was analyzed using Kaplan–Meier estimates and log-rank tests to compare the survival distributions of patients with and without these mutations. To control the false discovery rate (FDR) across multiple hypothesis testing scenarios in our genetic analyses, we employed the Benjamini–Hochberg procedure. This method adjusts for multiple comparisons by controlling the FDR at a threshold of 5%, providing a robust means to limit type I error. This is especially pertinent given our study’s extensive analysis of numerous genetic variants, ensuring that the reported associations are statistically significant and reducing the likelihood of false positives. A *p*-value of less than 0.05 was considered statistically significant. All statistical analyses were performed using Python (version 3.8) with relevant statistical libraries.

## 3. Results

### 3.1. Patient Demographics and Clinical Characteristics

A total of 51 patients with CMML from our center were included in this study. The cohort was divided into two groups: CMML-1 (n = 34) and CMML-2 (n = 17). A further category of patients (included in the group of 51 patients) undergoing AML transformation was analyzed (n = 14). The median age at diagnosis was 71 years (range: 35–88 years), with a predominance of male patients (71%). A previous or concomitant malignancy was diagnosed in 39% of the patients. Leukemic transformations occurred in 28% of the cohort. The median OS was significantly different between the groups, with 129 months for CMML-1, 29 months for CMML-2, and a significantly shorter survival for patients with CMML who went on to develop leukemic transformation (*p* = 0.0077) (Table 1).

### 3.2. Cytogenetic Analysis

The cytogenetic analysis revealed the following common abnormalities: monosomy 7, deletion 5q, trisomy 8, and complex karyotype (defined as three or more chromosomal abnormalities). Patients with monosomy 7 had a significantly shorter median OS than those without this abnormality. The median OS for patients with monosomy 7 was 14 months, while it was 42 months for those without (*p* < 0.05). Deletion 5q was observed in two patients. One patient had a survival of 15 months, and the other had a survival of 5 months. Both cases involved additional cytogenetic abnormalities. Due to the low number of cases, statistical analysis was not performed specifically for 5q deletions alone. Trisomy 8 was associated with significantly longer OS. The median OS for patients with trisomy 8 was 129.5 months compared to 44 months for those without (*p* < 0.05). Patients with a complex karyotype had the poorest survival outcomes. The median OS for these patients was 5 months compared to 45 months for patients with a normal karyotype or less complex abnormalities (*p* < 0.01).

### 3.3. Genetic Mutation Analysis

**Figure 1:**
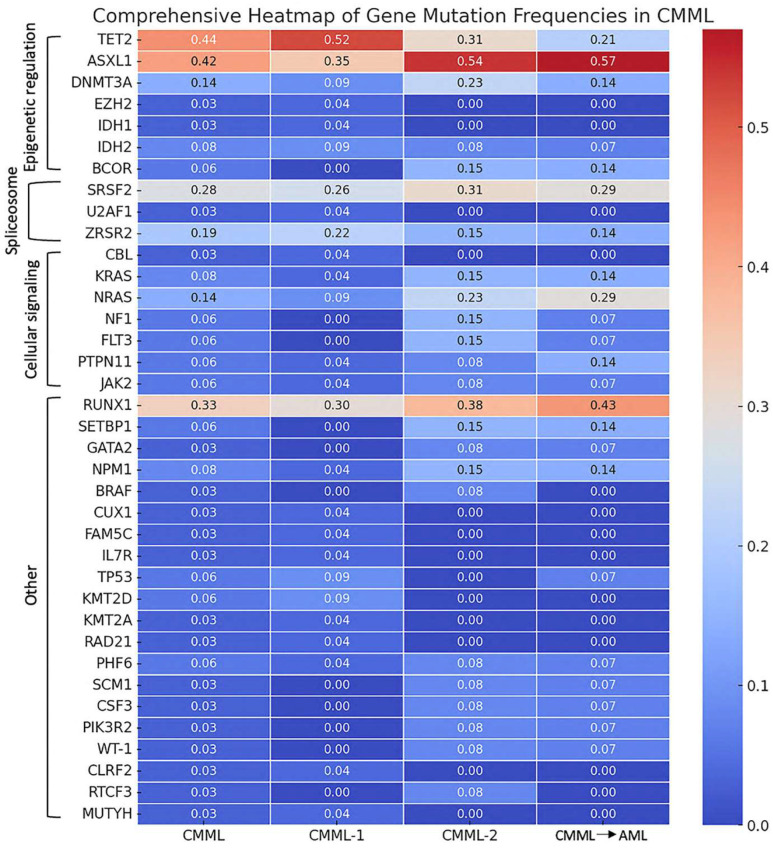
Heatmap of gene mutation frequencies in CMML. This heatmap illustrates the prevalence of various gene mutations across different stages and transformations of CMML. The data are categorized into three groups: CMML-1 (n = 23), CMML-2 (n = 13), and CMML, which later underwent transformation (n = 14), representing the progression stages of the disease.

The analysis of genetic mutations related to epigenetic regulation revealed significant variability across the patient groups. The TET2 mutation was present in 44% of the overall CMML cohort, with the highest frequency observed in CMML-1 patients (52%), followed by CMML-2 (31%). Patients who later transformed had a lower rate of TET2 mutations (21%). ASXL1 mutations were found in 42% of the total cohort, showing notable frequencies of 35% in CMML-1, 54% in CMML-2, and 57% in the transformation cohort. DNMT3A mutations were detected in 14% of patients, with similar occurrences across CMML-1 (9%), CMML-2 (23%), and AML transformation (14%). EZH2 mutations were rare, observed in only 3% of the total cohort, and exclusively found in CMML-1 patients (4%) (see Table 1 and Figure 1).

Mutations affecting the spliceosome machinery also showed distinct patterns. The SRSF2 mutation was the most common spliceosome-related mutation, found in 28% of the overall cohort, with 26% in CMML-1, 31% in CMML-2, and 29% in AML transformation. The ZRSR2 mutation was present in 19% of patients, with frequencies of 22% in CMML-1, 15% in CMML-2, and 14% in AML transformation. U2AF1 mutations were rare, detected in only 3% of patients and exclusively in the CMML-1 group (4%) (see Table 1 and Figure 1).

The analysis of mutations in cellular signaling pathways and other genes revealed further variability. NRAS mutations were present in 14% of patients, with higher frequencies in CMML-2 (23%) and AML transformation (29%) compared to CMML-1 (9%). KRAS mutations were found in 8% of the cohort, with 4% in CMML-1, 15% in CMML-2, and 14% in AML transformation. Other notable mutations included CBL (3% overall, detected only in CMML-1), FLT3, and PTPN11 (each 6% overall with variable frequencies). Among other genes, RUNX1 mutations were highly prevalent, detected in 33% of the total cohort, with higher frequencies in CMML-2 (38%) and cases that later transformed (43%) compared to CMML-1 (30%). SETBP1 mutations were found in 6% of patients across all groups. NPM1 mutations were present in 8% of the cohort, with similar frequencies across all groups (see Table 1 and Figure 1).

### 3.4. Statistical Analysis of Mutation Frequencies

The statistical analysis comparing mutation frequencies between CMML-2 and the other stages (CMML-1 and AML transformation) revealed that the TET2 mutation showed a trend towards significance when compared with CMML-1 (*p* = 0.065). No gene mutations showed significant differences compared to CMML-2 and AML transformation stages. These results indicate the potential role of TET2 in transitioning from CMML-1 to CMML-2. However, further research with larger datasets is needed to confirm this trend and validate these findings.

### 3.5. Survival Analysis and Outcomes in CMML Patients as Retrieved from the Kentucky Cancer Registry

**Figure 2:**
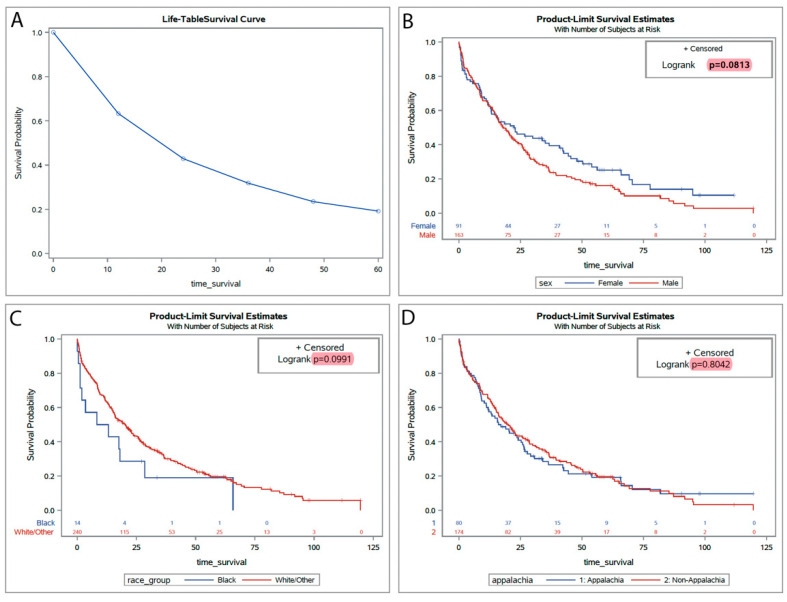
Kaplan–Meier survival estimates for CMML patients stratified by demographic and geographic variables. (**A**) Statewide Kaplan–Meier survival curves comparing localized hospital-based outcomes with broader statewide trends. (**B**) Survival analyses stratified by gender, highlighting potential differences in outcomes. (**C**) Survival analyses stratified by race provide insights into demographic influences on CMML outcomes. (**D**) Comparison of survival probabilities between patients in Appalachian versus non-Appalachian regions, assessing regional disparities in treatment outcomes.

We expanded our analysis by incorporating statewide population data to construct Kaplan–Meier plots and life table estimates, examining survival probabilities within a geographically and demographically diverse patient cohort. This statewide data from 2012 to 2021 offers insights into potential variations in treatment outcomes and survival rates, stratified by gender and race, thereby enriching our understanding beyond our hospital-specific dataset (Figure 2A). The mean age in this cohort was 74 years (ranging from 47 to 97). The broader dataset revealed no significant differences in survival when stratified by gender, with a log-rank *p*-value of 0.0813, suggesting marginal disparities between male and female patients in survival times across Kentucky. Racial analysis indicated slight variances in survival probabilities between Black and White/other racial groups, though these differences were not statistically significant (log-rank *p* = 0.0991) (Figure 2B,C). Furthermore, analysis considering Appalachian status showed no significant survival difference between Appalachian and non-Appalachian populations (log-rank *p* = 0.8042), reinforcing the need to explore other social and biological determinants that may influence disease outcomes (Figure 2D). Finally, the age-adjusted incidence of CMML in Kentucky (0.49/100,00) was not different from that of the rest of the United States (0.50/100,000, *p* = 0.596), as estimated based on SEER 17 registry data [4].

**Figure 3:**
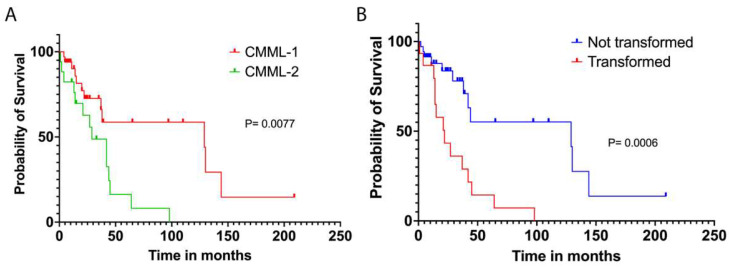
Kaplan–Meier survival curves for CMML patients at the University of Kentucky. (**A**) Survival probabilities between CMML-1 patients (red line) and CMML-2 patients (green line). (**B**) Survival probabilities for CMML patients, comparing those who have transformed (red line) versus those who have not (blue line). The *p*-values indicate the statistical significance of the differences in survival distributions (*p* = 0.0006 for transformation comparison, *p* = 0.0077 for subtype comparison).

Contrasting these findings with the Kaplan–Meier survival curves for CMML patients at the University of Kentucky, we observed pronounced differences in survival outcomes by clinical factors. The median OS for the University of Kentucky cohort was 42 months. Notably and not unexpectedly, there was a significant survival discrepancy between CMML-1 and CMML-2 subtypes, with median OS rates of 129 months and 29 months, respectively (*p* = 0.0077) (Figure 3A). Additionally, non-transformed patients at our institution displayed a median survival of 129 months, indicating superior outcomes compared to patients who later transformed, who had a median survival of 22 months (*p* = 0.0006) (Figure 3B). This disparity underscores the variance in disease progression and treatment response between the subtypes.

**Figure 4:**
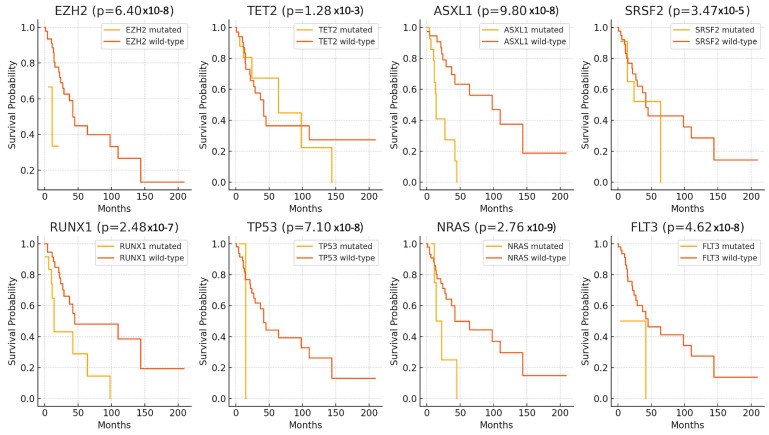
Kaplan–Meier survival curves for significant gene mutations in CMML patients. Kaplan–Meier survival curves for selected gene mutations significantly associated with survival outcomes in patients with CMML. Each panel represents a different gene, comparing survival probabilities over time between patients with mutations (yellow line) and wild-type (orange line) alleles.

In addition, the Kaplan–Meier survival analysis was employed to elucidate the impact of selected significant gene mutations on the survival outcomes of patients diagnosed with CMML. The analysis demonstrated that mutations in EZH2, TET2, ASXL1, SRSF2, RUNX1, TP53, NRAS, and FLT3 are associated with significantly decreased patient survival probabilities. Specifically, mutations in EZH2 (*p* = 6.40 × 10^−8^, TET2 (*p* = 1.28 × 10^−3^), ASXL1 (*p* = 9.80 × 10^–8^), SRSF2 (*p* = 3.47 × 10^–5^), RUNX1 (*p* = 2.48 × 10^–7^), TP53 (*p* = 7.10 × 10^–8^), NRAS (*p* = 2.76 × 10^–9^), and FLT3 (*p* = 4.62 × 10^–8^) were all linked to poorer survival outcomes compared to their respective wild-type counterparts (Figure 4). These findings suggest that these genetic alterations may serve as important prognostic markers in the studied cohort.

## 4. Discussion

CMML presents significant heterogeneity, both in clinical manifestation and in outcomes, predominantly affecting an older, primarily male population. Our study confirms these demographics, with median ages of 71 and 74 years in our institution and the broader Kentucky cohort, respectively. These findings are consistent with global research, which shows similar age and gender distributions. However, our data also highlight unique regional characteristics that may influence disease progression and treatment responses, underscoring the complexity of managing CMML.

A noteworthy aspect of our study is the examination of the referral patterns and their impact on patient characteristics and survival. Approximately 39% of our cohort had a history of other malignancies, a common feature among older populations, which, intriguingly, did not alter prognostic outcomes in our research. This aspect raises questions about the underlying mechanisms that may insulate survival outcomes from the impacts of comorbidities, suggesting a potential area for deeper investigation.

Survival rates at our institution are on par with or better than those reported by other academic centers and the state cancer registry, which could be attributed to the strategic use of demethylating agents and allogeneic transplantation. These therapies, while effective, highlight the need for tailored treatment approaches to further improve patient outcomes. The transformation rate into a blast phase or acute myelogenous leukemia in our cohort was 28%, comparable to 24% in Barcelona [5] and varying between 13% and 39% in Vienna [6], depending on the age at diagnosis. This transformation significantly shortens patient lifespans and underscores the urgency of developing strategies to mitigate this risk.

In the realm of genetic insights, our study brings to light the utility and limitations of current prognostic scores. Published scores incorporating mutations in RUNX1, NRAS, SETBP1, and ASXL1 provide a framework for assessing prognosis in CMML patients [7]. Additionally, similar analytical methodologies have been applied to evaluate outcomes post-allogeneic transplantation [8]. However, these prognostic scores often lack the precision necessary to guide treatment decisions for individual patients, reflective of the intrinsic variability within CMML pathogenesis.

This heterogeneity is further underscored by our genetic findings, where the mutation patterns within our Kentucky cohort revealed lower frequencies of TET2 mutations (44% vs. 60–73%) and higher frequencies of RUNX1 mutations (33% vs. 15–27%) compared to global data [1,5,9]. These discrepancies may stem from varying sensitivities of NGS technologies, differences in referral patterns, or inherent biological differences within the Kentucky population. Such variability suggests that while global data provide valuable benchmarks, local contextualization is crucial for optimal patient management.

Moreover, the confirmatory role of ASXL1 and NRAS mutations in influencing CMML prognosis, consistent with broader literature, emphasizes the potential of targeted therapies and highlights the need for enhanced predictive tools. Despite advancements, the prognosis for CMML has not seen substantial improvement over the past few decades. The adoption of treatments such as Venetoclax, though promising, underscores an ongoing need for novel therapeutic approaches and more refined prognostic tools [10,11,12].

The findings from the recent study on p53 mutations can be directly correlated with the clinical implications of TP53 mutations in CMML. In both solid and hematologic malignancies, including CMML, TP53 mutations are associated with poor prognosis, reduced overall survival, and a higher risk of progression to AML. The study’s demonstration of thermal instability in mutant p53 proteins, such as R248Q, R248W, and R273H, highlights a mechanism that contributes to oncogenesis by promoting the unfolding and aggregation of the p53 protein [13]. This results in the loss of its tumor-suppressive functions, consistent with the observed effects of TP53 mutations in CMML. The reduced DNA-binding ability of mutant p53 and its tendency to form aggregates may further explain the impaired tumor suppression in CMML, leading to more aggressive disease progression and resistance to therapeutic interventions.

The introduction of artificial intelligence (AI) in prognostication, incorporating a broad spectrum of biological, social, and clinical data, presents a significant opportunity. Recent advancements, as shown in myelodysplastic syndromes, suggest that AI could substantially improve the accuracy of risk stratification [14], potentially revolutionizing treatment paradigms in CMML.

Our findings resonate with those reported by Coston et al., who observed suboptimal response rates to hypomethylating agent therapy in CMML at a single institution (Table 2) [15]. This underscores the challenge of treating CMML and the urgent need for developing more effective therapeutic strategies responsive to the unique genetic profiles of different patient subgroups. Furthermore, the work by Pleyer et al. offers a broader view by detailing the outcomes of CMML patients treated with non-curative therapies in a retrospective cohort study. Their findings highlight the limited efficacy of current treatment modalities and the variability in patient survival, which parallels our observations of diverse clinical outcomes within our cohort [16].

While our findings provide valuable insights into the genetic underpinnings of CMML, the generalizability of these results may be limited by the study’s single-institution nature and the relatively small sample size. Future research could benefit from multi-center studies that include larger and more diverse patient populations. Such studies would not only validate our findings, but also potentially reveal additional insights that could be obscured in smaller, less varied cohorts.

## 5. Conclusions

In summary, while our study affirms several established characteristics of CMML, it also provides new insights into the genetic and clinical diversity within specific populations like Kentucky. These insights advocate for continued research into the molecular mechanisms of CMML and the development of advanced, precise treatment modalities that can profoundly enhance patient care and outcomes. Future studies should aim to further these findings, leveraging local and global data to foster a more nuanced understanding of CMML’s complex landscape.

## Figures and Tables

**Table 1:** Clinical and genetic features and subsequent events in 51 patients with CMML.

Variable	CMML (n = 51)	CMML-1 (n = 34)	CMML-2 (n = 17)	*p*-Value
Age in years; median (range)	71 (35–88)	73 (35–88)	66 (53–80)	0.0647
Male; *n* (%)	36 (71)	25 (74)	11 (65)	0.532
Other malignancies; *n* (%) ^a^	20 (39)	14 (41)	6 (35)	0.7672
Leukemic transformations; *n* (% LT)	14 (28)	4 (12)	10 (59)	0.0008
Overall survival; median months	42	129	29	0.0077
**Genetic Analysis**	**CMML** **(n = 36)**	**CMML-1** **(n = 23)**	**CMML-2** **(n = 13)**	**AML** **(n = 14)**
Epigenetic regulation analysis; n (%)				
TET2	16 (44)	12 (52)	4 (31)	3 (21)
ASXL1	15 (42)	8 (35)	7 (54)	8 (57)
DNMT3A	5 (14)	2 (9)	3 (23)	2 (14)
BCOR	2 (6)	0 (0)	2 (15)	2 (14)
EZH2	1 (3)	1 (4)	0 (0)	0 (0)
Spliceosome mutational analysis; n (%)				
SRSF2	10 (28)	6 (26)	4 (31)	4 (29)
*ZRSR2*	7 (19)	5 (22)	2 (15)	2 (14)
U2AF1	1 (3)	1 (4)	0 (0)	0 (0)
Cellular signaling analysis; n (%)				
NRAS	5 (14)	2 (9)	3 (23)	4 (29)
KRAS	3 (8)	1 (4)	2 (15)	2 (14)
CBL	1 (3)	1 (4)	0 (0)	0 (0)
FLT3	2 (6)	0 (0)	2 (15)	1 (7)
PTPN11	2 (6)	1 (4)	1 (8)	2 (14)
Other; n (%)				
RUNX1	12 (33)	7 (30)	5 (38)	6 (43)
SETBP1	2 (6)	0 (0)	2 (15)	2 (14)
NPM1	3 (8)	1 (4)	2 (15)	2 (14)

^a^ Patients with other cancers: prostate cancer (3), breast cancer (3), non-Hodgkin lymphoma (2), multiple cancers (2), and treatment-related cancers (2). Note on treatment: no treatment (11 patients), hydroxyurea (11 patients), decitabine (12 patients), azacitidine (11 patients), venetoclax with demethylating agents (2 patients), and various other protocols (4 patients). Special cases include 2 patients with treatment-related CMML and 5 who underwent allogeneic transplantation.

**Table 2:** Recent single- and multi-center institution studies for CMML.

Study Location	# of Patients	Median Age at Diagnosis (Years)	% Male	Timeframe	Median Survival (Months)	NGS Performed	Ref.
Lexington, KY (USA)	51	71	71	2005–2023	42	70.6%	This manuscript
Kentucky (USA)	254	73.9	64.2	2012–2022	23	NA	This manuscript
Rochester, MN	121	68	63	NA	~19	62%	[15]
38 centers worldwide	949	72	66	1981–2018	18.5	NA	[16]
Barcelona, SP	219	74.1	65.8	1997–2021	34	32.9%	[5]
Wuhan, CN	66	~65	63.0	2011–2020	10–18	25.8%	[17]
Bordeaux, FR	131	74	60	1999–2019	30–36	100%	[9]
Houston, TX (USA)	532	70	67%	2005–2022	35.9	70%	[18]

NA, not available.

## Data Availability

The datasets generated and analyzed during the current study are available from the corresponding author upon reasonable request. Additional data supporting this study’s findings are included within the article. All relevant data are contained within the manuscript, and high-resolution data are stored securely by the corresponding research institution and are available under specific conditions to researchers who meet the criteria for confidential data access.
